# Combined Effect of High-Pressure Processing with Spice Extracts on Quality of Low-Salt Sausage during Refrigerated Storage

**DOI:** 10.3390/foods10112610

**Published:** 2021-10-28

**Authors:** Qing Xiao, Mei Xu, Baocai Xu, Conggui Chen, Jieying Deng, Peijun Li

**Affiliations:** 1 China Light Industry Key Laboratory of Meat Microbial Control and Utilization, Hefei University of Technology, Hefei 230009, China; xiaoqing0715@163.com (Q.X.); baocaixu@163.com (B.X.); chencg1629@hfut.edu.cn (C.C.); dengjieying@hfut.edu.cn (J.D.); 2 School of Food and Biological Engineering, Hefei University of Technology, Hefei 230009, China; m13866107822_1@163.com

**Keywords:** high pressure processing, spice extracts, low-salt sausage, storage quality

## Abstract

The study evaluated the combined effect of high-pressure processing (HPP) and spice extracts on low-salt sausages during refrigerated storage. Physicochemical and microbiological characteristics of the sausages were determined. HPP treatment increased the thiobarbituric acid reactive substances (TBARS) value and the carbonyl content of the samples (*p* < 0.05), which meant lipid and protein oxidation was accelerated. Adding clove and cinnamon extracts can retard the oxidation caused by HPP (*p* < 0.05). The pH of the sausages treated with both the spice extracts and HPP maintained a higher pH value during the storage (*p* > 0.05). Compared with the samples treated with HPP or with the spice extracts alone, the combined treatment observably inhibited the growth of spoilage bacteria (*p* < 0.05) and improved the microbial community. The results demonstrated that the use of clove and cinnamon extracts in conjunction with HPP improved the storage quality and prolonged the shelf-life of the low-salt sausages. Thus, the combined use of spice extracts and HPP can be developed as a promising way to preserve low-salt meat products.

## 1. Introduction

According to the recommendation of the World Health Organization (WHO), the maximum level of sodium intake is 2 g, equivalent to 5 g of salt (NaCl) per day for adults. However, the sodium intake among many people exceeds the recommended level worldwide [1]. A high consumption of dietary salt is one of the causes of hypertensive disease, which, if left untreated, can lead to more serious cardiovascular diseases [2]. Researchers have demonstrated that nearly 80% of the daily salt intake comes from processed food, and meat products account for 20% of this total [3]. Therefore, implementing salt reduction strategies in the meat industry has become a focal point of research [4].

In meat products, salt plays pivotal roles in preventing microbial growth, conferring characteristic flavor and creating the desired texture [5,6,7]. Due to these, the reduction in salt may lead to considerable changes in the meat’s quality [8]. Nowadays, researchers have attempted to reduce the salt content by substituting NaCl with other chloride salts, such as KCl, CaCl_2_ and MgCl_2_ [9,10]. Among them, KCl is a highly valued alternative because it possesses similar functions to NaCl [11]. In addition, the potassium supplementation can contribute a protective effect against cardiovascular diseases [12]. Nevertheless, the partial substitution of NaCl with KCl generally has negative consequences on the storage quality of meat products [9]. Therefore, it is necessary to develop a new strategy that can improve this.

High pressure processing (HPP), a non-thermal processing technology, is considered as a green preservation method [8,13]. It has been used to extend the shelf-life of processed food with minimal impact on the sensory properties [14]. Previous researchers have indicated that applying HPP can enhance the water holding capacity, textural attributes and cooking yield of low-salt meat products [15,16]. It was also found that HPP treatment can increase the saltiness perception of meat products due to the weakening of the interaction between metal ions and proteins [15,17]. More importantly, HPP treatment can inactivate microorganisms and, thus, prolong the shelf-life of meat products [16]. It is reported that the degree of microbial damage caused by high pressure increased with the increase in pressure [18]. However, the negative effect caused by HPP, especially at a high-pressure level, is a problem. For example, HPP treatment can induce an alteration of the components in meat, such as lipids and proteins, and favor oxidation by promoting the formation of radicals [19]. Additionally, it has been proven that the HPP can increase the oxidation rate of meat products [20,21]. Furthermore, the costs of high-pressure equipment increase exponentially with operating pressures, which limits its use for an industrial procedure [22]. Therefore, it is of great importance to develop a combined method composed of lower-pressure treatment and other methods for improving the storage characteristics of low-salt meat products.

In recent years, studies have reported on natural antimicrobial substances, among which spice extracts provide unlimited opportunities for controlling microbial growth and inhibiting protein and lipid oxidation [23,24]. Clove and cinnamon, commonly used in meat products, were both found to exhibit antibacterial and antioxidant activities [25]. Chemical compounds such as eugenol and cinnamaldhyde are found in these essential oils and confirmed as the major chemical components responsible for exerting the antimicrobial and antioxidant activities [26]. A lot of researchers have indicated that clove and cinnamon can reduce the final microbial load and retard oxidation during chicken and fish meat storage [27,28]. Thus, considering the promotion of oxidation and high cost by HPP at high pressure levels, it is essential to clarify the effect of a combined use of HPP and spice extracts on the storage attributes of low-salt meat products. However, to our knowledge, there are few studies focused on this.

Since most microorganisms cannot be cultured in vitro, the culture-dependent method can only be recovered in a small percentage of the microorganisms present in poultry products. High-throughput sequencing (HTS) in determining meat quality has allowed for a quantitative in-depth assessment of changes in the microbial ecology during meat storage [29], and it can generate thousands of sequences within a short time to cover the complex microbial diversity and low-abundance microorganisms [30]. Therefore, HTS was applied to determine the microbial community in low-salt sausages.

The aim of the study was to determine the effect of HPP and spice extracts, either alone or in conjunction, on the quality of low-salt sausages during storage.

## 2. Materials and Methods

### 2.1. Preparation of Spice Extracts

Spice extracts were prepared according to Sun, Zhao, Chen, Zhang and Kong [26] with slight modifications. Clove and cinnamon were purchased from a local supermarket (Hefei, China) and dried at 60 °C for 24 h before grinding through an ultrafine pulverizer. After passing through an 80-mesh sieve, 50 g of spice powders was extracted with 500 mL of 85% ethanol at 60 °C for 24 h. The mixture was suction-filtered, and the filtered residue was extracted twice; then, the combined filtrates were evaporated on a rotary evaporator (RE-52AA, Yarong Biochemical Analysis Co., Ltd., Shanghai, China) at 55 °C in order to remove the ethanol. The two extracts were freeze-dried, ground into powder and stored at 4 °C until use.

### 2.2. Preparation of Low-Salt Sausage

Fresh chicken breast and pork back fat were purchased from a local supermarket (Hefei, China). Sausages were prepared using the following formulation with minor modification [26]. The chicken breast and pork back fat were ground separately through a meat grinder, and then were mixed evenly at a proportion of 3:1 (*w*/*w*). The minced meat was added with 1.75% NaCl (*w*/*w*), 0.75% KCl (*w*/*w*), 0.5% polyphosphates (*w*/*w*), 0.5% sugar (*w*/*w*) and 10% (*v*/*w*) ice water. The samples were either added with 0.05% clove and 0.05% cinnamon extracts or not. The resultant mixtures were mixed thoroughly and stuffed tightly into plastic casings 3.2 cm in diameter after being cured at 4 °C for 16 h. The sausages were cooked at 80 °C for 30 min in a thermostat water bather (HH-2, Lichen Bangxi Apparatus Co., Ltd., Shanghai, China). After cooling, the sausages were cut into 0.5-centimeter-thick slices in a sterile environment and subsequently vacuum packed in sterile bags (CYD002, Qingdao Hope Bio-Technology Co., Ltd., Qingdao, China). Then, the samples were treated with or without HPP in a 0.6-liter capacity 600 MPa high-pressure vessel (Baotou Kefa High-Pressure Technology Co., Ltd., Baotou, China) at 400 MPa for 10 min before storage.

Four groups of low-salt sausages were performed as follows: (1) Control (without any treatment), (2) SE (Treatment with 0.05% clove + 0.05% cinnamon extracts), (3) HPP (Treatment with HPP at 400 MPa for 10 min), (4) SE-HPP (Treatment with 0.05% clove + 0.05% cinnamon extracts and HPP at 400 MPa for 10 min). All the samples were stored in a refrigerator (5 ± 1 °C). The sausages were sampled for analysis at 0, 4, 8, 12, 16, 20 and 24 d during storage.

### 2.3. Measurement of pH Value

A total of 5 g of the sausage sample was homogenized with 45 mL of distilled water and then the mixture was filtered using through a 0.22-micrometer filter paper. The pH value of the filtrate was measured using a pH-meter (FE28, Mettler Toledo, Columbus, OH, USA).

### 2.4. Color Measurement

The color of the sausage sample was evaluated with a colorimeter (WSF, INESA., Virginia, Shanghai, China) in reflectance mode as described by Li, Kong, Qian, Zheng and Ning [31]. The minced sausage sample was tiled at the bottom of sample tanks. The color was described using the L*-(lightness) and b*-(yellowness) values. The colorimeter was calibrated with a white standard plate (L* = 99.99, a* = −0.20, b* = 0.23) before use.

### 2.5. Determination of Thiobarbituric Acid Reactive Substances (TBARS) Value

Lipid oxidation was analyzed using the thiobarbituric acid reactive substances (TBARS) method described by Jayawardana, Liyanage, Lalantha, Iddamalgoda and Weththasinghe [32] with minor modifications. A total of 5 g of the sausage sample was homogenized with 50 mL of a cold trichloroacetic acid (TCA) reagent (7.5% TCA, 0.1% EDTA) at 3000 rpm for 1 min. Afterwards, the mixture was filtered (0.22-micrometer filter paper) and the resultant filtrate (5 mL) was added with equal volume of thiobarbituric acid (0.02 mol/L). After incubation at 90 °C for 30 min, the mixture was cooled to 25 °C and the absorbance was determined at 532 nm using a microplate reader (H1, Boten Instrument Co., Ltd., Winooski, VT, USA). 1,1,3,3-Tetraethoxypropane was used as a positive control for standard curve. 

### 2.6. Measurement of Carbonyl Content

Carbonyl contents of the sausage samples were determined by a reaction with 2,4-dinitrophenyl hydrazine (DNPH) according to the method of Berardo, Maere, Stavropoulou, Rysman, Leroy and Smet [33] with slight modifications. A total of 3 g of the sausage sample was homogenized in 30 mL of phosphate buffer (20 mM, pH 6.5, containing 0.6 M NaCl) at 10,000 rpm for 15 min. The mixture was divided into four aliquots with 0.2 mL each. All of the four aliquots were treated with 1 mL of 10% cold TCA. After centrifugation (8000× *g*, 10 min), the supernatant was discarded, and the two aliquots were treated with 0.5 mL of 10 mM DNPH (*w*/*v*) in 2.0 M HCl, while another two aliquots were treated with 0.5 mL of 2.0 M HCl (blank). After a 1-hour reaction, 0.5 mL of TCA (20%) was added. The samples were centrifuged at 12,100× *g* for 5 min and the supernatant was discarded. Excess DNPH was removed by washing three times with 1 mL of ethanol and ethyl acetate solution (1:1, *v*/*v*). The pellets were resuspended in 1 mL of 6.0 M guanidine hydrochloride with phosphate buffer (20 mM, pH 6.5). The carbonyl content (nmol/mg protein) was calculated using the following equation:

(1) $$\frac{C_{\mathrm{hydrazone}}}{C_{\mathrm{protein}}}=\frac{A_{370}}{\varepsilon_{\mathrm{hydrazone},370}\times (A_{280}-A_{370}\times 0.43)}\times 10^{6}$$

where *ε*_hydrazone,__370_ is 2.2 × 10^4^ M^−1^·cm^−1^, and A_370_ and A_280_ represent absorbance at 370 and 280 nm, respectively.

### 2.7. Microbiological Analysis

#### 2.7.1. Microbial Counts

The bacteria counts were measured using the method of Yong, Liu, Xiong, Xiao, Hu and Wang [30]. Total viable counts (TVC) were analyzed on Plate Count Agar (Guangdong Huan-kai Microbial Sci. & Tech. Co, Ltd., Guangzhou, China) incubated at 37 °C for 48 h. Enumeration of Lactic acid bacteria (LAB) was conducted on de Man Rogosa and Sharpe Agar (Guangdong Huan-kai Microbial Sci. & Tech. Co, Ltd., Guangzhou, China) after a 48-hour incubation at 37 °C under anaerobic conditions. Brochothrix *thermosphacta* counts were counted using STAA Agar Medium (Qingdao Hope Bio-Technology Co., Ltd., Qingdao, China) after a 48-hour incubation at 25 °C. *Clostridium perfringens* was enumerated on Tryptose Sulfite-Cyloserine Agar Base (Qingdao Hope Bio-Technology Co., Ltd., Qingdao, China) incubated at 37 °C for 48 h under anaerobic conditions. 

#### 2.7.2. High-Throughput Sequencing

Total bacterial DNA was extracted from the sausage samples using a GenElute™ Kit (Tiangen Biotech, CO., Ltd., Beijing, China) according to the manufacturer’s instructions. A total of 0.5 g of sausage samples were added to 1 mL of CTAB buffer solution (Real-Times Biotechnology Co., Ltd., Beijing, China) containing lysozyme before being incubated with shaking for 60 min in a 65 °C water bather. All samples were centrifuged at 4000× *g* for 30 min; after that, the supernatant was added with 800 µL of the mixture (phenol:chloroform:isoamyl alcohol mixture = 25:24:1) (*w*/*v*/*v*). The resultant mixtures were centrifuged to obtain a precipitate, which were subsequently added to 2.5 mL of isopropanol. The precipitated DNA was harvested by centrifuging at 13,800× *g* for 10 min, washed twice with 1 mL of 75% ethyl alcohol and dried in air. The DNA was re-dissolved in 50 µL of double distilled water (containing ribonuclease A) and stored at −20 °C until use. PCR amplifications of the V4 region of bacterial 16S rRNA gene were carried out using the primer pairs 515F (5′-GTGCCAGCMGCCGCGGTAA-3′) and 806R (5′-GGACTACHVGGGTWTCTAAT-3′). The PCR reactions were carried out in a 30-microliter reaction system as described by the method [34]. Then, the purified amplicons of all the samples were used to generate sequencing libraries with the NEB Next^®^ Ultra™ DNA Library Prep Kit for Illumina (New England Biolabs, Ipswich, MA, USA) according to the manufacturer’s recommendations and analyzed using an Illumina HiSeq platform (Illumina, San Diego, CA, USA).

### 2.8. Statistical Analysis

All specific experiments were carried out in triplicates and the data were expressed as the mean ± SD values. The results were subjected to analysis of variance using the Statistix 8.1 software package (Analytical Software, St. Paul, MN, USA). Differences between treatments were analyzed using the least significant difference test, and significance was defined at *p* < 0.05. Significant differences (*p* < 0.05) among means were identified using Tukey’s honest significant difference procedure.

## 3. Results and Discussion

### 3.1. pH Value

**Figure 1:**
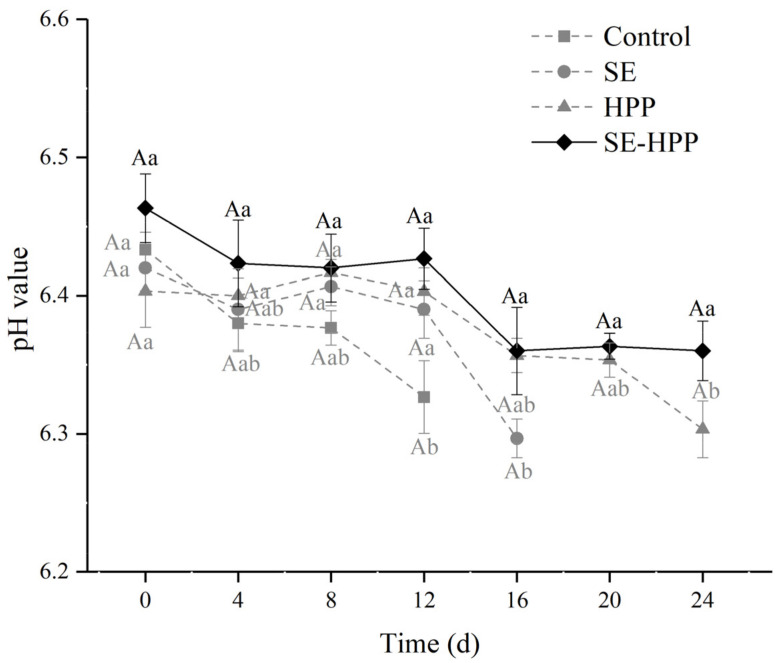
Effect of spice extracts and HPP, either alone or in conjunction, on the pH values of low-salt sausages during storage. Means with uppercase letter A indicate no difference among different treatments at the same time points (*p* < 0.05) and means with different lowercase letters (a–b) indicate significant differences among different times for the same treatments (*p* < 0.05).

The pH value is an important parameter in monitoring meat quality. There was no significant change in the pH value of all the samples during the first 8 d (Figure 1). At 12 d, the pH value of the control group was significantly lower compared with the beginning of storage (*p* < 0.05), which may be attributed to the organic acid accumulation produced by spoilage microorganisms [35]. It was noticed that among the three groups, the pH value of SE group was the lowest at 16 d. Researchers have shown that HPP can inhibit the growth of spoilage and pathogenic bacteria in various meat products [36]. At 24 d, the pH value of the HPP group showed a significant decrease compared with the storage initial stage (*p* < 0.05). Meanwhile, the pH value of the SE-HPP group was close from 0 d to 24 d, indicating the possible existence of a synergistic antimicrobial effect of the spice extracts and HPP treatment.

### 3.2. Color

The effects of SE and HPP on the L* value and b* value of low-salt sausages in refrigerated storage are presented in Table 1. Compared with the control and HPP groups, the L* values of the SE and SE-HPP groups were significantly lower during the entire storage period (*p* < 0.05). It has been found that the L* value diminished in the cooked beef patties with clove extract due to a purple pigment [37]. In the high pressure processed chicken sausage, a similar phenomenon was discovered as well due to the pressure-induced modifications of sarcoplasmic and myofibrillar proteins [38].

The b* value of the HPP samples was close to that of the control groups during storage, while the SE and SE-HPP samples displayed higher b* values (*p* < 0.05). The b* value of meat products can be significantly increased by high-pressure treatment [39,40]. This phenomenon is likely due to enzymatic deactivation and protein denaturation for the change in yellowness [41]. Furthermore, Zhang, Wu and Guo [11] found that the b* value of samples treated with spice extracts were higher than the control, which may be affected by the slight color compounds in the spice extracts.

### 3.3. TBARS Values

**Figure 2:**
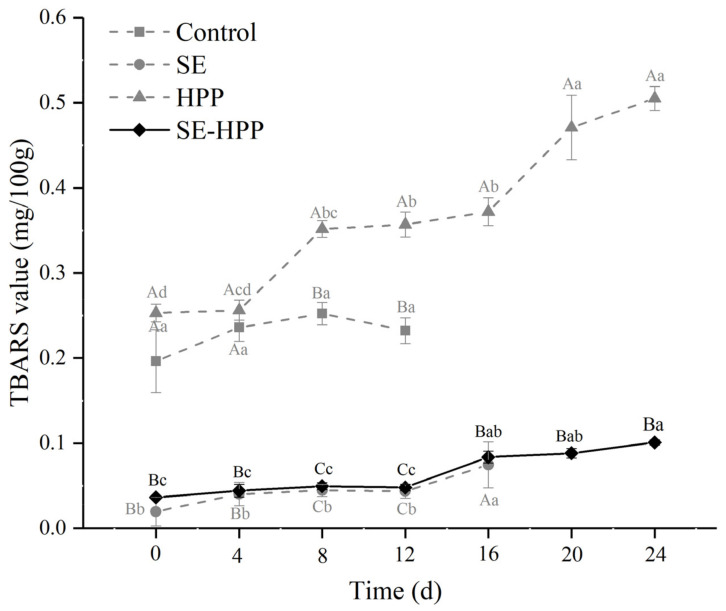
Effect of spice extracts treatment combined with HPP, either alone or in conjunction, on the TBARS values of low-salt sausage during storage. Means with different uppercase letters (A–C) indicate significant differences among different treatments at the same time points (*p* < 0.05) and means with different lowercase letters (a–d) indicate significant differences among different times for the same treatments (*p* < 0.05).

The TBARS value is used to determine the formation of secondary lipid oxidation products, such as malondialdehyde (MDA). Compared with the control and HPP groups, the TBARS values of the SE and SE-HPP groups were at a lower level during the whole storage period (Figure 2). The main antioxidative components of clove and cinnamon extract are eugenol and cinnamaldehyde, respectively [42,43]. It has been reported that eugenol has a 90% antioxidant activity such as that of Betahydroxyl anisole (BHA) [44]. Additionally, the clove extract showed a high 2,2-diphenyl-1-picrylhydrazyl (DPPH) radical scavenging activity [28]. Cinnamaldehyde is the major ingredient in cinnamon extract, which has been used as an antioxidant in food preservation [34]. Previous results also found that adding cinnamon essential oils markedly decreased the peroxide values and delayed oxidation in beef [45].

In this study, the TBARS value of the HPP group exceeded that of the control group after 8 d (*p* < 0.05). This was consistent with the results of Khan, Ali, Yang and Kamboh [41], who found HPP (400 MPa, 10 min) treatment accelerated the lipid oxidation in duck breast meat. HPP may cause lipid oxidation in meat products [46]. Under high pressures, heme proteins undergo conformational changes, leading to the release of non-heme iron and thereby promoting the lipid oxidation [47,48]. Furthermore, the radicals were found to be formed in meat during the HP treatment (400 MPa), which conduced the initiation of lipid oxidation [49].

At 12 d, the TBARS value of the control sample displayed a decreasing trend, which may be due to the binding between MDA and protein degradation products [47]. For the HPP treated low-salt sausage, the TBARS value increased from 0.25 to >0.50 mg/100 g during the 24-day storage, indicating that the lipid oxidation was markedly increased with the storage time. Excessive lipid oxidation may contribute to the off flavor of chicken meat products. It has been suggested that the meat quality deteriorated, supposing that the content of MDA exceeded 0.5 mg/100 g [50]. Compared with the results of the control and HPP sample, it was found that the addition of the spice extracts can not only delay the lipid oxidation of the low-salt sausages, but also compensate for the oxidation caused by HPP treatment. Therefore, the integration of spice extracts and HPP treatment is an effective strategy to improve meat products’ storage quality.

### 3.4. Carbonyl Content

**Figure 3:**
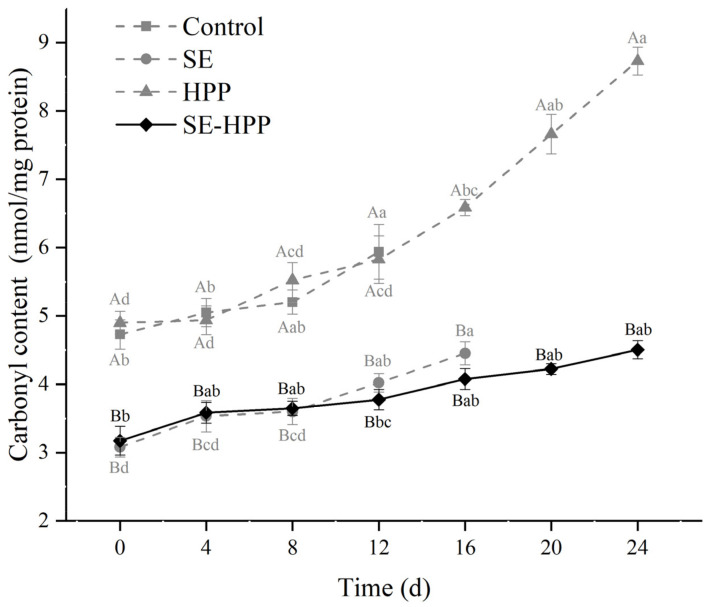
Effect of spice extracts treatment and HPP, either alone or in conjunction, on the carbonyl content of low-salt sausages during storage. Means with different uppercase letters (A–B) indicate significant differences among different treatments at the same time points (*p* < 0.05) and means with different lowercase letters (a–d) indicate significant differences among different times for the same treatments (*p* < 0.05).

As shown in Figure 3, the carbonyl contents of all the groups increased gradually during storage. The HPP-treated samples had the highest carbonyl content of up to 7.89 nmol/mg during the storage (*p* < 0.05). The carbonyl contents in the SE and SE-HPP groups were significantly less than those of the control and HPP groups throughout the whole storage (*p* < 0.05). This indicated that the SE and SE-HPP treatment can effectively retard the protein oxidation, which was consistent with the results of lipid oxidation in these samples, as described in Section 3.3. Furthermore, there was no significant difference between the SE and SE-HPP groups during storage (*p* > 0.05).

Protein oxidation caused by high-pressure processing has been reported [51,52]. Protein carbonyls are mainly formed by means of the following three pathways: metal-catalyzed oxidation, non-enzymatic glycation and adduct formation with non-protein carbonyl compounds [40]. The third way of protein carbonylation is the interactions between protein and lipid oxidation products, such as MDA and 4-hydroxynonena. In this study, the control and HPP groups displayed higher MDA than the other two groups, indicating the enhancement of lipid oxidation, which can generate more free radicals. The radicals are potential initiators of protein carbonylation, leading to the protein oxidation [53]. Moreover, free radicals released by lipid oxidation can also attack amino acid side chains, resulting in protein oxidation [54].

Antioxidants can prevent chain reaction, scavenge active oxygen molecules and chelate metal ions, which can inhibit the carbonyl formation [55]. Chen, Diao, Li, Chen and Kong [56] observed that clove extracts inhibited the formation of carbonyl compounds in pork meat, which was attributed to the ability of the eugenol to scavenge DPPH radical and chelate Fe^2+^. Thus, it was concluded that the spice extracts can effectively inhibit the protein oxidation caused by the HPP treatment. Additionally, the combination of spice extracts and HPP treatment can be used to improve the storage quality of the low-salt sausages.

### 3.5. Microbial Analysis

#### 3.5.1. Microbial Community Using the Culture-Dependent Method

**Figure 4:**
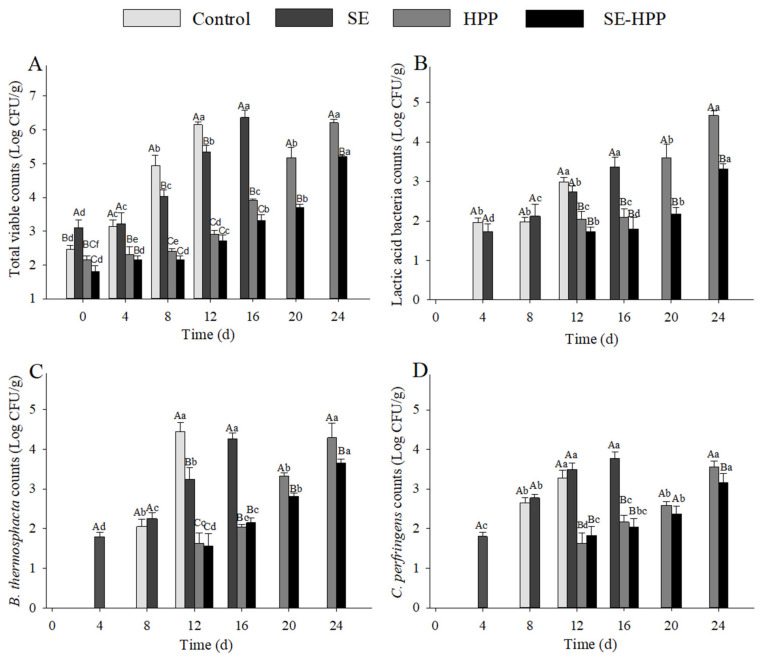
Effect of spice extracts and HPP, either alone or in conjunction, on the total viable counts (**A**), lactic acid bacteria counts (**B**), *B. thermosphacta* counts (**C**) and *C. perfringens* counts (**D**) of low-salt sausages during storage. The data were not reported if the TVC for a sample exceeded 5 Log CFU/g. Means with different uppercase letters (A–C) indicate significant differences among different treatments at the same time points (*p* < 0.05) and means with different lowercase letters (a–f) indicate significant differences among different times for the same treatments (*p* < 0.05).

The changes in TVC and counts of LAB, *B.* *thermosphacta* and *C. perfringens* of the low-salt sausages are shown in Figure 4A. At the initial stage, the TVC of the SE group was significantly higher than the other groups (*p* < 0.05), which may be due to the spice extracts containing high level microorganisms, and the cooked temperature (80 °C) for the sausages was insufficient to kill all the organisms [57]. At 8 d, lower TVC were observed in the SE group (*p* < 0.05) compared with the control group, which may be related to the antimicrobial activity of the spice extracts. In fact, eugenol and cinnamaldehyde can cause irreversible damage to cell membranes, resulting in a leak of the cell content and a decrease in intracellular enzyme activity [58].

During the first 12 d of storage, the TVC of the control and SE samples were over 5 Log CFU/g, a threshold value for cooked meat products [59]. Then, lower TVC (less than 3 Log CFU/g) were observed in the HPP and SE-HPP groups (*p* < 0.05). It has been reported that high-pressure treatment can be fatal for most of the microorganisms by affecting the DNA replication and transcription, increasing the membrane permeability and destroying the enzyme system [60,61]. A minimum level of 400 MPa is known to be sufficient to induce a remarkable microbial reduction in meat products [62]. After 24 d of storage, the TVC of the HPP and SE-HPP sample were 6.37 and 5.27 Log CFU/g, respectively, which exceeded 5 Log CFU/g, indicating that an integration of the spice extracts and HPP treatment displayed a synergetic bacteriostatic effect on the low-salt sausages.

The LAB behavior in response to HPP and spice extracts in low-salt sausages has rarely been investigated. The initial LAB counts in all the groups were not detected (less than 1 Log CFU/g; Figure 4B), and there was no significant difference between the control and the SE group during the 12-day storage (*p* > 0.05). For the HPP and SE-HPP groups, the LAB counts were still not found until storage for 12 d. As the storage time increased to 24 d, the LAB counts in the SE-HPP group were significantly lower than that in the HPP group (*p* < 0.05). High-pressure treatment may cause sublethal damage for some of the LAB at the beginning of storage. The bacteria could recover and outgrow during the late period of storage. Additionally, then, the growth was restricted as a result of the antibacterial activity of clove and cinnamon extracts [63]. Similar to this result, it has been suggested that Gram-positive bacteria are more susceptible to spice extracts than Gram-negative bacteria [64]. Thus, the combination of the HPP and spice extracts resulted in a synergy that considerably impaired the microbial recovery caused by the HPP treatment.

*B. thermosphacta* was the main spoilage microorganism in chicken meat treated with HPP [65]. At 8 d, the *B. thermosphacta* counts in the control and SE groups were 2.05 and 2.25 Log CFU/g, respectively (Figure 4C). The bacterium still could not be detected in the other groups until 12 d. During the late storage period, the SE-HPP group showed a lower level in *B. thermosphacta.* Karam, Chehab, Osaili and Savvaidis [66] have also verified that treatment with spice extracts can significantly reduce *B. thermosphacta* counts (*p* < 0.05). Less than 1 Log CFU/g of *B. thermosphacta* was noticed in sliced vacuum-packed cooked ham after high-pressure treatment [67].

Chicken meat is recognized as the main source of *C. perfringens*, which is one of the most prevalent foodborne pathogens associated with gas gangrene and food poisoning [68]. As shown in Figure 4D, the counts of *C. perfringens* in the SE group reached approximately 1.8 Log CFU/g after 4 d of storage, while none was detected in the other groups. Previous studies have reported the genus *Clostridium* was considered as one of the predominant flora in spices [69]. The *C. perfringens* counts of the HPP and SE-HPP groups were significantly lower than the SE group after 16 d. It may be due to the fact that high-pressure treatment can inhibit the germination and outgrowth of the *C. perfringens* spores [36]. After 24 d of storage, the *C. perfringens* quantity in the HPP group reached 3.42 Log CFU/g and was significantly higher than that of the SE-HPP group (*p* < 0.05) It indicated that the spice extracts can effectively inhibit the *C. perfringens* growth during storage. Additionally, Lins and Philipp [70] have also found that cinnamon can effectively suppress the growth of *C. perfringens* spores. It concluded that spice extracts integrated with HPP treatment can synergically control the *C. perfringens* germination and outgrowth.

#### 3.5.2. Microbial Diversity Using High-Throughput Sequencing

**Figure 5:**
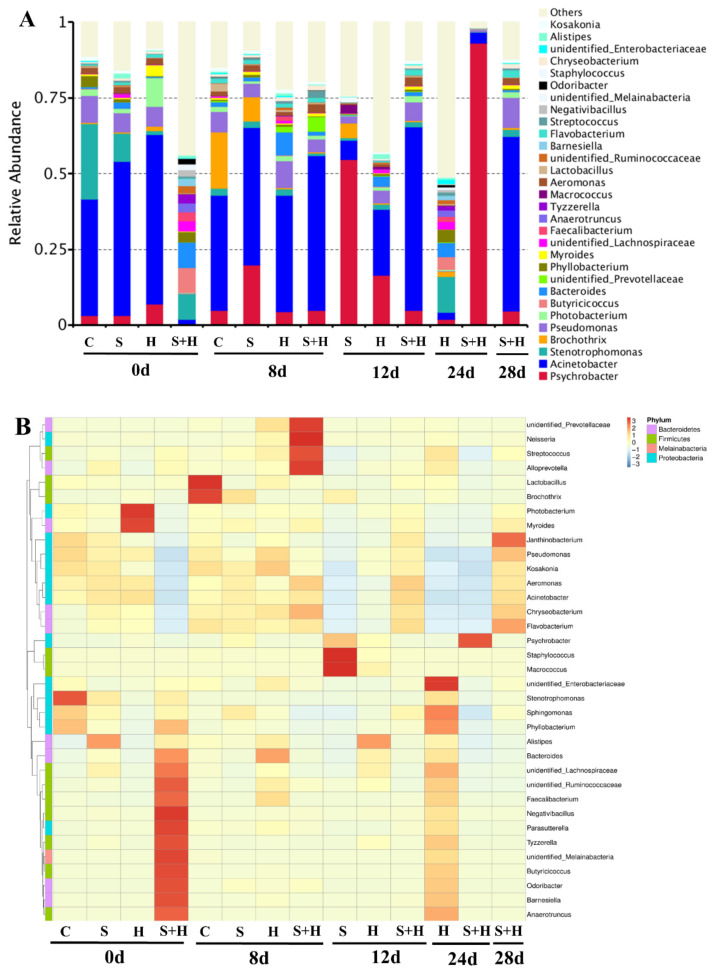
Effect of spice extracts and HPP, either alone or in conjunction, on the bacterial relative abundance (**A**) and hierarchically clustered heat map (**B**) at the genus level in low-salt sausages during storage. (C: Control group, S: SE group, H: HPP group, S + H: SE-HPP group).

As shown in Figure 5A, the microbial profiles were similar in the control group, SE group and HPP group at the beginning of the storage, where *Psychrobacter* spp., *Pseudomonas* spp. and *Acinetobacter* spp. dominated the flora, while *Stenotrophomonas* spp., *Butyricicoccus* spp. and *Bacteroides* spp. were found to be the predominant ones in the SE-HPP group. Compared with all the other groups, the control sample bacterial community was mainly composed of *Acinetobacter* spp. and *Brochothrix* spp. At 8 d. It was indicated that both the spice extracts and the HPP treatment inhibited the growth of *Brochothrix* spp. In the low-salt sausages, which was consistent with the result of the *B. thermosphacta* counts (Figure 4C). As shown in Figure 5B, the heatmap showed the changes in the microbial communities of all the groups. After 12 d of storage, *Psychrobacter* spp. Dominated in the SE group, with a relative abundance of 54.78%. *Acinetobacter* spp., accounting for 21.67%, was predominant in the HPP group, followed by *Psychrobacter* spp. (16.49%). For the SE-HPP group, *Acinetobacter* spp. accounted for 60.72% as the most predominant bacteria. The result indicated that both the HPP treatment and the spice extracts inhibited the growth of *Psychrobacter* spp. in the low-salt sausages.

After 24 d of storage, the diversity of Gram-negative bacteria in the HPP group increased remarkably. Meanwhile, the relative abundance of Gram-positive bacteria, including *Brochothrix* spp. and *Lactobacillus* spp., in the HPP group were significantly higher than that in the SE-HPP group (*p* < 0.05), which was in good agreement with the results of the *B. thermosphacta* and LAB counts (Figure 4). These results demonstrated that the combined use of spice extracts and HPP treatment exhibited synergistic effects on the spoilage bacteria.

## 4. Conclusions

The study determined the effect of HPP and spice extracts, either alone or in conjunction, on the quality of low-salt sausages during storage. Studies have shown that HPP treatment can lead to a reduction in the counts of spoilage bacteria but can also accelerate the lipid and protein oxidation in the low-salt sausages, which had a negative effect on the sensory quality of the products. Adding spice extracts can not only retard the oxidation, but also inhibit the growth of spoilage bacteria in the sausages. The results demonstrated that the use of spice extracts in conjunction with HPP improved the storage quality of the low-salt sausages. It provides a natural way for the preservation of low-salt meat products. In this study, KCl was used to make low-salt sausages by a partial substitution of NaCl. Future studies are required to focus on the natural preservation methods of low-salt sausages formulated with chloride salts.

## Figures and Tables

**Table 1:** Effect of spice extracts and HPP, either alone or in conjunction, on the color values of low-salt sausages during storage (5 °C). Means with different uppercase letters (A–C) indicate significant differences among different treatments at the same time points (*p* < 0.05) and means with different lowercase letters (a–b) indicate significant differences among different times for the same treatments (*p* < 0.05).

Treatment	Storage Time (d)						
	0	4	8	12	16	20	24
L* value							
Control	97.82 ± 0.07 ^Aa^	97.69 ± 0.03 ^Aa^	97.69 ± 0.04 ^Aa^	97.62 ± 0.07 ^Aa^			
SE	97.32 ± 0.05 ^BCa^	97.15 ± 0.08 ^BCab^	96.88 ± 0.09 ^Ca^	97 ± 0.14 ^Bab^	97.1 ± 0.14 ^Bab^		
HPP	97.6 ± 0.11 ^ABa^	97.45 ± 0.07 ^ABa^	97.61 ± 0.04 ^Aa^	97.53 ± 0.17 ^Aa^	97.42 ± 0.17 ^Aa^	97.46 ± 0.1 ^Aa^	97.7 ± 0.05 ^Aa^
SE-HPP	97.07 ± 0.25 ^Ca^	96.94 ± 0.23 ^Ca^	97.22 ± 0.09 ^Bb^	96.78 ± 0.14 ^Ba^	96.76 ± 0.09 ^Ca^	96.92 ± 0.01 ^Ba^	96.9 ± 0.11 ^Ba^
b* value							
Control	37.2 ± 0.84 ^Ba^	37.01 ± 0.4 ^Ba^	36.63 ± 1.0 ^Ba^	37.85 ± 0.52 ^Ba^			
SE	37.87 ± 0.66 ^Bb^	39.88 ± 1.2 ^ABab^	41.04 ± 1.16 ^Aa^	41.58 ± 0.68 ^Aa^	42.71 ± 0.61 ^Aa^		
HPP	38.4 ± 0.27 ^Ba^	38.09 ± 1.18 ^ABa^	36.09 ± 1.01 ^Bab^	36.82 ± 1.77 ^Ba^	37.8 ± 1.44 ^Ba^	37.23 ± 0.82 ^Ba^	32.91 ± 0.6 ^Ba^
SE-HPP	41.6 ± 0.66 ^Aa^	43.4 ± 3.21 ^Aa^	43.18 ± 0.99 ^Aa^	44.43 ± 0.88 ^Aa^	45.8 ± 1.19 ^Aa^	42.86 ± 0.11 ^Aa^	43.64 ± 0.83 ^Aa^

## Data Availability

Datasets generated during the current study are available from the corresponding author on reasonable request.
